# Morphological description and near-complete mitochondrial genome of *Haemoproteus (Parahaemoproteus) trarotraro* n. sp.: a widely distributed species reported in Brazilian falcons

**DOI:** 10.7717/peerj.20653

**Published:** 2026-02-24

**Authors:** Lis Marques de C. Vieira, M Andreina Pacheco, Ananias A. Escalante, Erika M. Braga

**Affiliations:** 1 Department of Parasitology, Universidade Federal de Minas Gerais, Belo Horizonte, Minas Gerais, Brazil; 2 Biology Department/Institute of Genomics and Evolutionary Medicine, Temple University, Philadelphia, PA, United States of America

**Keywords:** Haemosporida, Falconiformes, Wildlife rehabilitation center, Species definition

## Abstract

Haemosporida are vector-borne parasitic protozoa known to be present in birds of most avian orders. However, despite their perceived diversity using DNA barcode approaches, describing and delimiting species is challenging, particularly for those parasites found in non-passerine birds. In this study, we describe *Haemoproteus trarotraro* n. sp., a species found in two Falconiform hosts, the Crested Caracara (*Caracara plancus plancus*, type host) and the Yellow-headed Caracara (*Daptrius chimachima chimachima*), both sampled in Brazil at a wildlife rehabilitation center using microscopy and molecular tools. * Haemoproteus trarotraro* n. sp. is distinguished from the two other haemoproteid species described in Falconiformes, *H. brachiatus* and * H. tinnunculi* , by the absence of gametocytes that fully encircle the host-cell nucleus, and by the presence of numerous small vacuoles scattered throughout the cytoplasm of macrogametocytes. Both the partial *cytb* gene and the mtDNA genome for this new species are reported. The sequencing of the *cytb* barcode fragment revealed that *H. trarotraro* n. sp. reported here corresponds to a *Haemoproteus* sp. haplotype (GenBank Accession (AF465594) lineage POLPLA01 in Malavi) previously reported from *Caracara plancus cheriway* in Florida, USA. Although it diverges by only  2% at the *cytb* level from *H. tinnunculi* and *H. brachiatus*, *H. trarotraro* n. sp. is not a sister lineage to these taxa. Instead, phylogenetic analyses place it within a distinct but closely related, well-supported clade comprising lineages infecting American Kestrels (*Falco sparverius*). This study contributes, through an integrative taxonomic approach, to the ongoing discussion about species delimitation within the order Haemosporida.

## Introduction

Haemosporida are a diverse order of heteroxenous vector-borne parasites that infect a wide range of vertebrate hosts (Garnham, 1966; Valkiūnas, 2005; Atkinson, Thomas & Hunter, 2008). They have been particularly well studied in birds, since they occur in nearly all avian families and their species radiation seems to follow the diversification of modern birds (Van Riper et al., 1986; Atkinson et al., 1995; Valkiūnas, 2005; Fecchio et al., 2014; Fecchio et al., 2018; Pacheco et al., 2018a; Fecchio et al., 2021; Fecchio et al., 2022; Penha et al., 2022; de Angeli Dutra et al., 2023; Pacheco & Escalante, 2023; Tamayo Quintero et al., 2025). Parasites of the three genera commonly found in avian hosts, *Plasmodium*, *Haemoproteus*, and *Leucocytozoon,* are present in all terrestrial ecosystems, and some species can cause severe disease (Van Riper et al., 1986; Atkinson et al., 1995; Cardona, Ihejirika & McClellan, 2002; Valkiūnas, 2005; Galvão Bueno et al., 2010; Vanstreels et al., 2014a; Sijbranda et al., 2017; Hernandez-Colina et al., 2021; Galen et al., 2022). Among them, *Haemoproteus*—particularly the subgenus *Parahaemoproteus*—stands out as the most diverse within the order Haemosporida and is transmitted primarily by biting midges (Diptera: Ceratopogonidae) (Valkiūnas, 2005; Valkiūnas & Iezhova, 2022). Although long considered benign, growing evidence has highlighted the pathogenic potential of *Haemoproteus* infections, particularly in naïve bird species (Cardona, Ihejirika & McClellan, 2002; Pacheco et al., 2011; Tostes et al., 2015; Ortiz-Catedral et al., 2019; Groff et al., 2019; Hernández Lara et al., 2021; Yoshimoto et al., 2021; Galen et al., 2022; Salem et al., 2025).

Although passerines remain the most extensively studied hosts of avian haemosporidians, there is a growing recognition of the need to sample additional avian orders to better characterize parasite diversity across the avifauna (Vieira et al., 2023; Hennig et al., 2024; Vieira et al., 2025a). Among these, the order Falconiformes—which currently comprises 65 recognized species (Gill, Donsker & Rasmussen, 2023)—harbors a considerable diversity of haemosporidian parasites (Outlaw & Ricklefs, 2009; Valkiūnas, 2005). However, only two *Haemoproteus* morphospecies are currently considered valid for this order: *H. tinnunculi* Wasielewski and Wülker, 1918 and *H. brachiatus* Valkiūnas and Iezhova, 1989, both described in Eurasian Kestrel (*Falcon tinnunculus*) from the Holarctic region (Valkiūnas, 2005; Valkiūnas & Iezhova, 2022). Due to their diversity and widespread distribution (Fuchs, Johnson & Mindell, 2012), falconiform birds serve as valuable models for investigating parasite diversity (Kutzer, Frey & Kotremba, 1980). Several species are considered vulnerable and affected by anthropogenic activities, making them the focus of conservation programs, including efforts to rescue and treat individuals in wildlife rehabilitation centers (Souza, Vilela & Câmara, 2014). These centers may facilitate pathogen spillover, as diverse avian species are housed in close proximity without prior pathogen screening or vector control (Galvão Bueno et al., 2010; Vanstreels et al., 2014a; Sijbranda et al., 2017; Hernandez-Colina et al., 2021).

In this context, we sampled two falconiform species admitted to the Wildlife Screening Center of Belo Horizonte (CETAS-BH) in Brazil: The Crested Caracara (*Caracara plancus*) and the Yellow-headed Caracara (*Daptrius chimachima*). Infection with a *Haemoproteus* POLPLA01 lineage previously reported in *C. plancus* from the United States (Ricklefs & Fallon, 2002) was identified in both host species. Through integrative taxonomy—combining morphological characterization with molecular data—we describe a new species, *Haemoproteus trarotraro* n. sp. Although genetically similar lineages have been reported from other Falconiformes (Outlaw & Ricklefs, 2009; Outlaw & Ricklefs, 2010; Tinajero et al., 2019), Strigiformes (Ishak et al., 2008), Coraciiformes (Fecchio et al., 2019), and Passeriformes (Durrant et al., 2006) across the Americas, this is the first report of this parasite in falcons from Brazil.

## Materials & Methods

### Ethical statement

This research was approved by the Ethics Committee on Animal Experimentation (CETEA) of the Universidade Federal de Minas Gerais, Brazil (Protocol No. 48/2024), and by the Instituto Estadual de Florestas (IEF) under Authorization No. 75722467 (Process No. 2100.01.0035718/2023-91).

### Hosts’ description and sampling

The Crested Caracara (*Caracara plancus*) is a non-migratory, opportunistic raptor that inhabits open landscapes such as grasslands, pastures, and habitats associated with savanna and semi-arid biomes, including the Caatinga, an ecoregion characterized by semi-arid tropical vegetation in interior northeastern Brazil (Morrison & Dwyer, 2021). Its distribution ranges from the southern United States and northern Mexico to the southernmost regions of South America. *Caracara plancus* is currently divided into two subspecies: *Caracara plancus cheriway*, which inhabits the southern United States, parts of Central America, northern South America, and Cuba; and *Caracara plancus plancus*, found from eastern to southern South America (Dove & Banks, 1999; Morrison & Dwyer, 2021). In Brazil, the latter can be frequently observed, even in highly urbanized areas.

The Yellow-headed Caracara (*Daptrius chimachima*) shares similar habitat preferences with *C. plancus*, and their habitats overlap, particularly in South America. It is also a non-migratory species, distributed from southern Costa Rica to northern Argentina (Bierregaard et al., 2022). Two subspecies are recognized: *Daptrius chimachima cordatus*, which occurs from Central America through Colombia, the Guianas, Trinidad, and into the eastern Amazon region; and *Daptrius chimachima chimachima*, restricted to South American countries such as Brazil, Bolivia, Paraguay, Argentina, and Uruguay (Bierregaard et al., 2022).

Although *C. plancus* and *D. chimachima* both belong to the subfamily Polyborinae and exhibit similar ecological traits, their most recent common ancestors within the clade differ. Historically grouped within the genus *Polyborus*, recent molecular phylogenetic analyses have shown that the Yellow-headed Caracara’s closest relative is the Black Caracara (*Daptrius ater*), together forming a sister group to a clade including the Chimango Caracara (*Daptrius chimango*) and other former members of the genus *Phalcoboenus* (Fuchs, Johnson & Mindell, 2012). In contrast, *C. plancus* forms a monophyletic clade comprising its two subspecies, which is sister to a larger clade encompassing the genera *Ibycter*, the former *Phalcoboenus* and *Daptrius*, including *D. chimachima* (Fuchs, Johnson & Mindell, 2012; Prum et al., 2015). The subfamily Polyborinae forms a sister group to Falconinae, which includes other generalists and widely distributed falcons, such as those in the genus *Falco* (Fuchs, Johnson & Mindell, 2012; Prum et al., 2015).

Thirty-four Crested Caracaras and six Yellow-headed Caracaras were sampled at CETAS-BH, Minas Gerais, Brazil, between January 2024 and January 2025. These individuals had been rescued by environmental agencies, NGOs, or private citizens and brought to the facility, often injured or presenting with comorbidities. Notably, one Yellow-headed Caracara (ID 8) exhibited an oral *Trichomonas* sp. infection and the absence of one eyeball, while one Crested Caracara (ID 50) presented with mite infestation. All individuals underwent integrative taxonomic analysis, combining molecular diagnostics for haemosporidian parasites with morphological assessment through blood smear examination.

### Blood samples processing and morphometry

Samples were collected and processed as previously described by Vieira et al. (2025b). Specifically, upon arrival at the rehabilitation center, blood samples were collected for smear preparation and DNA extraction to ensure that infections had not been acquired during captivity. The smears were fixed in absolute methanol, stained with 10% Giemsa solution (pH 7.2), and thoroughly examined at 1,000 × magnification using an Olympus CX31 light microscope. Parasitemia levels were estimated by scanning 200 randomly chosen microscopic fields (Godfrey, Fedynich & Pence, 1987). Parasitological imaging and morphometric measurements were also carried out following Vieira et al. (2025b), and the criteria established by Valkiūnas (2005) and Bennett & Campbell (1972). Statistical procedures were performed using R software (version 4.4.2; R Foundation for Statistical Computing, Vienna, Austria).

### DNA extraction, *cytb* and mtDNA sequencing

DNA extraction was performed using the chelating resin method (5% Chelex 100), as described by Vieira et al. (2025b), including positive (*Plasmodium falciparum*) and negative (Milli-Q water) controls. The DNA concentration was measured using a NanoDrop 2000 spectrophotometer (Thermo Fisher Scientific, Waltham, MA, USA), and the samples were stored at –20 °C until further use.

*Cytb* 479 bp fragment amplification followed the *nested*-PCR protocols of Hellgren, Waldenström & Bensch (2004), using specific primers for *Haemoproteus/Plasmodium* spp. A volume of one µL of sample was used for both the first and second reactions. Both previously extracted controls and new functional controls were included to monitor reaction performance. PCR products were resolved with 6% polyacrylamide gels and visualized using silver nitrate staining. Amplified products were then purified *via* 20% polyethylene glycol 6000 (PEG) precipitation, following the method of Sambrook & Russel (2001), and sequenced bidirectionally at the Sanger sequencing facility of the René Rachou Institute –Fiocruz/MG. Resulting electropherograms were analyzed according to Vieira et al. (2025b) and compared against sequences available in GenBank (https://www.ncbi.nlm.nih.gov/) and the MalAvi database (Bensch, Hellgren & Pérez-Tris, 2009; Data sent by Staffan Bensch on 07/05/2025). The partial *cytb* gene sequences were submitted to the GenBank under the accession number PX209361 and PX209362.

Then, the positive Crested Caracara (ID 50) sample was used to amplify the near-complete parasite mtDNA genome using a nested PCR protocol with Takara LA Taq™ polymerase (TaKaRa Takara Mirus Bio, San Jose, USA; Pacheco et al., 2018b). Briefly, two independent PCRs were carried out in 50 µL using 3 µL of the total DNA and outer oligos forward AE170 5′-GAGGATTCTCTCCACACTTCAATTCGTACTTC-3′ and reverse AE171 5′-CAGGAAAATWATAGACCGAACCTTGG ACTC-3′. For the nested PCRs, 1 µL of the primary PCRs and the inner oligos forward AE176 5′-TTTCATCCTTAAATCTCGTAAC-3′ and reverse AE136 5′-GACCGAACCTTGGACTCTT-3′ were used. Negative (distilled water) and positive controls (*Plasmodium cynomolgi*) were included. Amplification conditions for both PCRs were an initial denaturation at 94 °C for 1 min and 30 cycles of 30 s at 94 °C and 7 min at 67 °C, followed by a final extension of 10 min at 72 °C. PCR products (50 µL) were excised from the gel (bands of ∼six kb), purified using the QIAquick Gel extraction kit (Qiagen, GmbH, Hilden, Germany), and cloned into the pGEM-T Easy Vector system (Promega, Madison, WI, USA). Both strands of the two clones were sequenced at Genewiz from Azenta Life Sciences (Burlington, MA, USA). Both clones were identical, suggesting that only one *Haemoproteus* species was found in this individual. The mtDNA genome sequence obtained in this study was identified as *Haemoproteus* sp. POLPLA01 lineage (haplotype 46: AF465594; Ricklefs & Fallon, 2002) using BLAST against MalAvi dataset (Bensch, Hellgren & Pérez-Tris, 2009) and GenBank (Benson et al., 2013). This mtDNA genome sequence was submitted to the GenBank under the accession number PX277568.

The electronic version of this article in Portable Document Format (PDF) will represent a published work according to the International Commission on Zoological Nomenclature (ICZN), and hence the new names contained in the electronic version are effectively published under that Code from the electronic edition alone. This published work and the nomenclatural acts it contains have been registered in ZooBank, the online registration system for the ICZN. The ZooBank LSIDs (Life Science Identifiers) can be resolved and the associated information viewed through any standard web browser by appending the LSID to the prefix http://zoobank.org/. The LSID for this publication is: urn:lsid:zoobank.org:pub:E3DAA6A6-1060-4192-A64E-B734CAB9C97C. The online version of this work is archived and available from the following digital repositories: PeerJ, PubMed Central SCIE and CLOCKSS.

### Phylogenetic analyses

To estimate the phylogenetic relationships between *Haemoproteus* sp. POLPLA01 lineage (haplotype 46: AF465594) sequence obtained here and previously reported haemosporidian *cytb* gene and mtDNA genome sequences, two alignments were constructed using MAFFT v.7 (Katoh & Standley, 2013) with manual editing. The first alignment was constructed using 94 haemosporidian partial *cytb* gene sequences (450 excluding gaps) from four Haemosporida genera (*Plasmodium, Haemocystidium, Haemoproteus,* and *Leucocytozoon*) available from GenBank (Benson et al., 2013) and MalAvi database (Bensch, Hellgren & Pérez-Tris, 2009). A second alignment was done using the near-complete parasite mtDNA genomes available in GenBank (Benson et al., 2013). This alignment included 146 mt sequences (5,082 bp excluding gaps) from five Haemosporida genera (*Plasmodium, Hepatocystis, Haemocystidium, Haemoproteus,* and *Leucocytozoon*) plus the sequence obtained here (ID sample 50, PX277568). In both cases, *Leucocytozoon* (*Leucocytozoon*) spp. sequences were chosen as an outgroup.

Next, phylogenetic hypotheses were estimated using both the Maximum Likelihood method as implemented in IQ-TREE v2.3.1 (Nguyen et al., 2014) and the Bayesian method executed in MrBayes v3.2.7 with the default priors (Ronquist & Huelsenbeck, 2003). A general time-reversible model with gamma-distributed substitution rates and a proportion of invariant sites (GTR + Γ + I) was used in both methods, as the best substitution model determined by ModelFinder in IQ-TREE (Nguyen et al., 2014; Kalyaanamoorthy et al., 2017). Bootstraps were generated through Ultrafast bootstrap approximation (UFBoot) using 1,000 replicates (Minh, Nguyen & von Haeseler, 2013) for the Maximum Likelihood tree. In the case of the Bayesian method, posterior probabilities (PP) for the nodes were estimated from two independent chains of 6 × 10^6^ Markov Chain Monte Carlo (MCMC) steps by sampling every 500 generations. Convergence was assumed when the value of the potential scale reduction factor (PSRF) was between 1.00 and 1.02, and the average standard deviation of the posterior probability was <0.01 (Ronquist & Huelsenbeck, 2003). A 25% “burn-in” of the sample was discarded. Phylogenetic trees were visualized using FigTree v1.4.4 (http://tree.bio.ed.ac.uk/software/figtree/). Avian parasite species names, GenBank accession numbers, lineage name, posterior probabilities and bootstraps for each node are shown in the phylogenetic tree figure.

## Results

### Parasite detection *via* microscopy and PCR

Out of the 40 individuals examined, only one Yellow-headed Caracara (individual ID 8) and one Crested Caracara (individual ID 50) tested positive for *Haemoproteus* sp., confirmed by both PCR/sequencing (lineage POLPLA01) and microscopy. Both were adult individuals, with no available information on the exact locations where they were rescued within the state of Minas Gerais, Brazil. During the rehabilitation process, the individuals who tested positive on the first day of sampling, corresponding to their arrival at CETAS (Day 0), underwent five additional collections to monitor the infection and increase the chances of morphological characterization of different blood stages of the parasites. Individual ID 8 was first sampled on February 1, 2024 (Day 0), with subsequent collections occurring on February 8, 2024 (Day 1), February 15, 2024 (Day 2), February 19, 2024 (Day 3), February 23, 2024 (Day 4), and March 11, 2024 (Day 5). Individual ID 50 was first sampled on March 5, 2024 (Day 0), with additional samples collected on March 8, 2024 (Day 1), March 11, 2024 (Day 2), March 15, 2024 (Day 3), April 4, 2024 (Day 4), and April 21, 2024 (Day 5) (Table 1).

**Figure 1:**
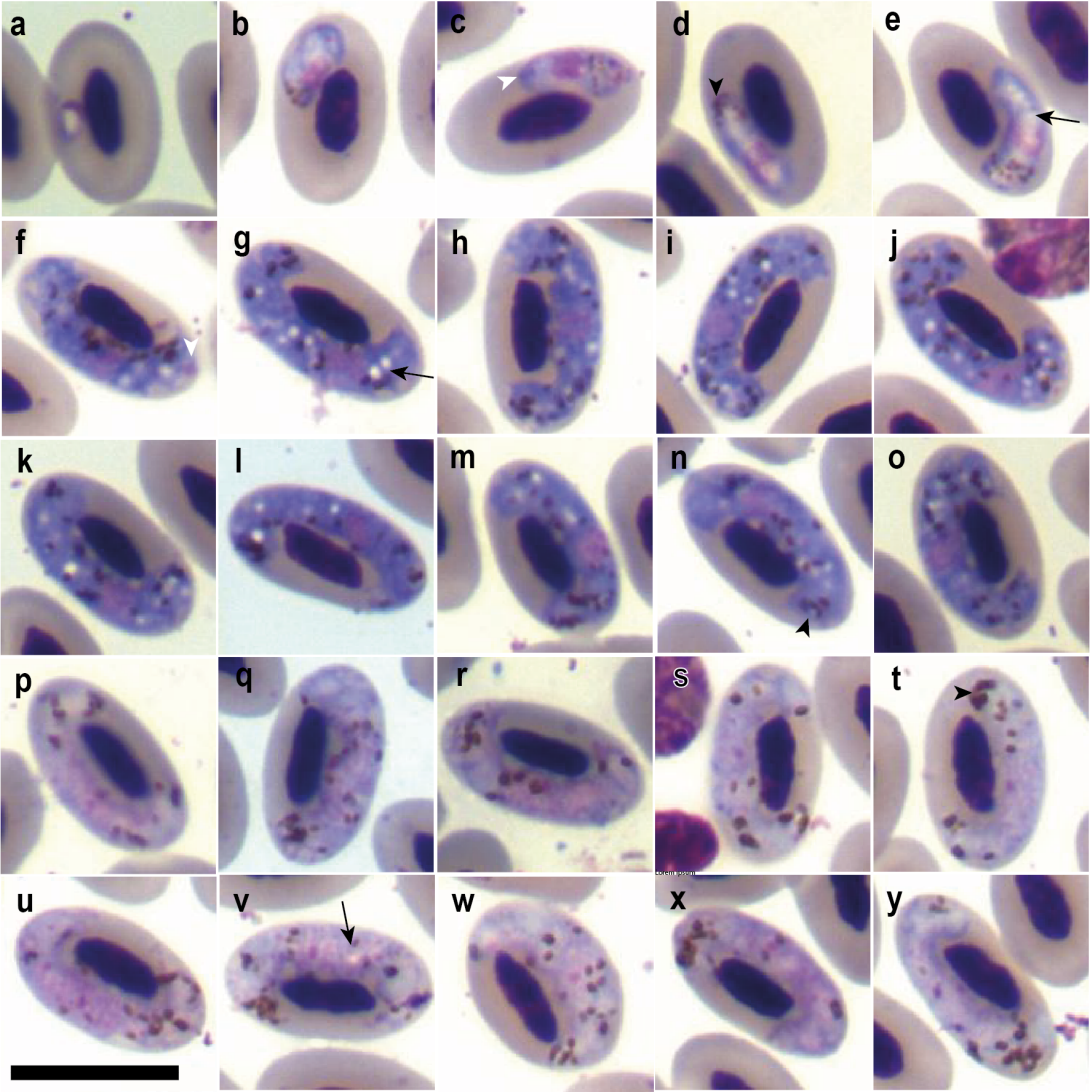
Gametocytes of *Haemoproteus trarotraro.* n. sp. from the blood of Crested Caracara (*Caracara plancus*) sampled in Minas Gerais, Brazil. (A–E) Young gametocytes. (F–O) Macrogametocytes. (P–Y) Microgametocytes. Black arrowheads: haemozoin granules; black long arrows: vacuoles; white arrowheads: volutin. Giemsa-stained thin blood films. Scale bar = 10 µm.

All positive individuals exhibited parasitemia levels adequate for detailed morphological characterization, with one Crested Caracara individual reaching parasite intensities exceeding 2% in circulating blood (Table 1). Both young and mature gametocytes were widely distributed throughout blood smears (Fig. 1). No evidence of coinfection or mixed infections was detected through either microscopic examination or electropherogram analysis of the partial *cytb* gene. The DNA sequences obtained from both individuals showed 100% identity with *Haemoproteus* sp. haplotype 46 (GenBank Acc. No. AF465594, linegae POLPLA01 in MalAvi database), which was previously identified in a Crested Caracara in Florida, USA (Ricklefs & Fallon, 2002). Notably, this lineage—and no other closely related lineages at the *cytb* level—had previously been associated with any morphological descriptions.

### Morphological description of *Haemoproteus (Parahaemoproteus) trarotraro* n. sp.

The forms found in both *C. plancus* (Fig. 1) and *D. chimachima* (Fig. S1) were indistinguishable, reinforcing the robustness of the morphological data used to describe this new species and further indicating that the same parasite lineage occurring in two different falconiform hosts did not exhibit notable phenotypic variation. The subgenera *Haemoproteus* and *Parahaemoproteus* cannot be distinguished by examining the gametocytes in the bloodstream. However, the biology of parasites in the subgenus *Haemoproteus* contributes to their specialization for infecting hosts in the order Columbiformes (Valkiūnas, 2005). Thus, since *H. trarotraro* n. sp. parasitizes Falconiformes, we considered its placement within the subgenus *Parahaemoproteus*, a finding that was later corroborated by molecular analyses.

**Table 1:** Changes in *Haemoproteus trarotraro* n. sp. parasitemia across multiple sampling days in different hosts.

**Host**	**Day 0**	**Day 1**	**Day 2**	**Day 3**	**Day 4**	**Day 5**
Crested Caracara *(Caracara plancus*)	0.05%	0.07%	0.09%	0.07%	2.1%	0.8%
Yellow-headed Caracara (*Daptrius chimachima*)	0.005%	0.007%	0.01%	0.006%	0.005%	0.5%

### Young gametocytes

Young gametocytes develop within mature erythrocytes, occupying the polar or subpolar region of the host cell (Figs. 1A–1E). They contain small hemozoin granules grouped at one pole of the parasite (Figs. 1A–1E) and display an accumulation of volutin primarily at the periphery of the parasite’s cytoplasm (Figs. 1A–1E). The parasites are vacuolated (Figs. 1A–1E) and have a rounded, centrally located nucleus (Figs. 1A–1E).

### Macrogametocytes

Macrogametocytes develop within mature erythrocytes, occupying one side and both poles of the host cell, without completely enveloping the host nucleus (Figs. 1F–1O). Nevertheless, in most cases, the parasite was in contact with the host nucleus, either fully—where the entire lateral surface of the nucleus was in contact with the parasite’s cytoplasm (Figs. 1F, 1G, 1O)—or partially, with cytoplasmic projections of the parasite contacting the host nucleus at discrete points (Figs. 1H, 1M, 1N). The parasite exhibited numerous hemozoin granules (average = 16.4; average length = 0.6 µm, Table 2) distributed throughout the cytoplasm, usually forming clusters in the polar region (Figs. 1F–1I, 1K–1N), along with abundant, uniformly dispersed volutin that imparts an intense coloration to the cytoplasm (Figs. 1F–1O). Multiple small vacuoles (average length = 0.6 µm, Table 2) were scattered within the cytoplasm (Figs. 1F–1O). The parasite’s nucleus is round, centrally located, and, in most forms, lacks a visible nucleolus (Figs. 1F–1O). It causes only a slight lateral displacement of the host cell nucleus but significantly alters the host cell’s dimensions (Table 2).

**Table 2:** Morphometric parameters of mature gametocytes of *Haemoproteus trarotraro* n. sp. and its host cells from the peripheral blood of Crested Caracara (*Caracara plancus*). Minimum and maximum values are provided, followed in parentheses by the arithmetic mean or median and standard deviation or median absolute deviation, depending on data normality.

**Features**	***H. trarotraro*** ** n. sp. from** ***C. plancus*** ** (μm)**
**Uninfected erythrocyte**	(***n***=**30**)
Lenght	11.9–13.7 (13.0 ± 0.5)
Width	6.8–8.4 (7.7 ± 0.4)
Area	65.9–88.5 (78.9 ± 4.7)
Nucleus lenght	5.6–7.0 (6.3 ± 0.4)
Nucleus width	2.3–3.3 (2.8 ± 0.2)
Nucleus area	12.3–16.7 (14.1 ± 1.0)
**Macrogametocytes**	(***n***=**26**)
Lenght	14.1–21.7 (17.5 ± 1.7)
Width	2.5–4.0 (3.2 ± 0.4)
Area	39.9–56.8 (47.8 ± 4.6)
Lenght of the nucleus	1.7–3.6 (2.3 ± 0.2)
Widht of the nucleus	1.2–2.4 (1.7 ± 0.3)
Area of the nucleus	1.5–5.1 (3.2 ± 0.8)
NDR	0.4–0.9 (0.7 ± 0.1)
Number of hemozoin granules	12.0–22.0 (16.4 ± 2.7)
Lenght of hemozoin granules	0.4–0.9 (0.6 ± 0.0)
Lenght of vacuoles	0.3–1.0 (0.6 ± 0.1)
Lenght of infected host cell	12.2–15.6 (13.9 ± 0.9)
Widht of infected host cell	6.9–9.2 (7.6 ± 0.5)
Area of infected host cell	72.8–95.4 (84.4 ± 5.4)
Lenght of infected host cell nucleus	5.4–7.1 (6.2 ± 0.5)
Width of infected host cell nucleus	2.3–3.0 (2.6 ± 0.1)
Area of infected host cell nucleus	11.0–15.2 (12.9 ± 1.2)
**Microgametocytes**	(***n***=**31**)
Lenght	13.9–23.1 (19.0 ± 2.4)
Width	2.0–5.0 (3.2 ± 0.3)
Area	45.0–61.0 (51.5 ± 4.3)
NDR	0.3–1.1 (0.6 ± 0.1)
Number of hemozoin granules	8.0–20.0 (16.0 ± 3.1)
Lenght of hemozoin granules	0.4–1.0 (0.7 ± 0.1)
Lenght of infected host cell	11.7–15.7 (13.9 ± 0.9)
Widht of infected host cell	6.5–9.3 (7.8 ± 0.6)
Area of infected host cell	74.3–94.5 (85.5 ± 5.4)
Length of infected host cell nucleus	5.7–7.1 (6.4 ± 0.4)
Width of infected host cell nucleus	2.2–3.2 (2.6 ± 0.2)
Area of infected host cell nucleus	10.9–17.0 (13.8 ± 1.2)

### Microgametocytes

They exhibit the common dimorphic characteristics reported for the order Haemosporida, such as paler staining and a loosely compact nucleus within the cytoplasm (Figs. 1P–1Y). Like the macrogametocytes, they occupy a lateral position relative to the host cell nucleus, with the parasite’s poles curving around the poles of the host nucleus but not completely encircling it. The amount and size of hemozoin granules is likewise comparable (Table 2); however, accumulations of hemozoin granules at one pole of the parasite are more frequently observed than in macrogametocytes (Figs. 1P, 1Q, 1T, 1V, 1X). They often display cytoplasmic projections that make contact with the host nucleus at certain points (Figs. 1Q, 1S, 1U, 1V, 1Y). In contrast to macrogametocytes, which may completely adhere to the nucleus in a lateral position, small gaps between the parasite cytoplasm and the host nucleus are usually present (Figs. 1P–1Y). They exhibited larger dimensions in both length and area relative to macrogametocytes; however, they presented decreased NDR and equivalent distortion effects on host cell morphology (Table 2). The presence of vacuoles was rare (Fig. 1V) and possibly mistaken with depigmented cytoplasmic areas. Due to the lack of definition of the parasite’s nucleus and vacuoles, their measurements were not included in the morphometric data.

### Remarks

*Haemoproteus trarotraro* n. sp. differs markedly from *H. brachiatus* and *H. tinnunculi* by the absence of gametocyte forms that completely or nearly completely encircle the host cell nucleus, as well as by the presence of numerous small vacuoles distributed throughout the cytoplasm of macrogametocytes (Figs. 1F–1O). Although it presents amoeboid projections, these are subtle, and no finger-like forms—commonly observed in *H. brachiatus*—were detected. Additionally, *H. trarotraro* n. sp. exhibits intense cytoplasmic staining due to the presence of volutin granules, a feature not observed in *H. brachiatus.*

### Taxonomic summary

***Type host***: The Crested Caracara, *Caracara plancus* (Falconidae, Falconiformes).

***Additional hosts***: Lineages with more than 99% identity at the *cytb* level have been repeatedly reported in *Falco sparverius* (Falconidae, Falconiformes) (FALC7, GenBank Acc. No. GQ141621, and FALC11, GenBank Acc. No. GQ141558, Outlaw & Ricklefs, 2009; AMKE_DR49, GenBank Acc. No. GU251991, and RP02_CRC2, GenBank Acc. No. GU252008, Outlaw & Ricklefs, 2010; *Haemoproteus* sp. isolate F57, GenBank Acc. No. MF621945, Tinajero et al., 2019). However, records also exist in the Barn Owl, *Tyto alba* (Strigidae, Strigiformes) (BAOW5465, GenBank Acc. No. EU627830, Ishak et al., 2008), the Golden-billed Saltator, *Saltator aurantiirostris* (Thraupidae, Passeriformes) (Haemoproteus sp. U48, GenBank Acc. No. DQ241555, Durrant et al., 2006), and the *Green Kingfisher*, *Chloroceryle americana* (Alcedinidae, Coraciiformes) (CHLAME01, GenBank Acc. No. MK695448, Fecchio et al., 2019). It is important to note that the lineages reported from non-Falconiform hosts are most likely abortive infections. Therefore, the apparent host range of *H. trarotraro* should be interpreted with caution.

***Type locality***: Minas Gerais, Brazil (18°17′44″S, 52°45′02″W).

***Additional localities***: Ricklefs & Fallon (2002) reported the same lineage in Florida, USA (*Haemoproteus* sp. haplotype 46, GenBank Acc. No. AF465594, POLPLA01 lineage in MalAvi database). Lineages with more than 99% identity at the *cytb* gene level have also been reported from different localities in the USA: FALC7 (GenBank Acc. No. GQ141621) and FALC11 (GenBank Acc. No. GQ141558) (Outlaw & Ricklefs, 2009); RP02_CRC2 (GenBank Acc. No. GU252008) and AMKE_DR49 (GenBank Acc. No. GU251991) (Outlaw & Ricklefs, 2010); and BAOW5465 (GenBank Acc. No. EU627830) (Ishak et al., 2008). In Latin America, records exist from Mexico (isolate F57, GenBank Acc. No. MF621945; Tinajero et al., 2019), Uruguay (*Haemoproteus* sp. U48, GenBank Acc. No. DQ241555; Durrant et al., 2006), and Brazil (CHLAME01, GenBank Acc. No. MK695448; Fecchio et al., 2019). The locality range should also be interpreted with caution, as birds sampled in those areas might have been paratenic.

***Type specimen***: Hapantotype blood slides (lineage 050, Acc. No. PX209361 infection intensity = 2.1%, *Caracara plancus*, Belo Horizonte, Minas Gerais, Brazil, April 2024) were deposited in the Hemoparasite Collection of the Federal University of Minas Gerais (Coleção de Hemoparasitos da Universidade Federal de Minas Gerais –UFMG-HEM), Belo Horizonte, Brazil, under the accession number UFMG-HEM-0058 (lineage 050). Parahapantotype blood slides (lineage 008, Acc. No. PX209362 infection intensity = 0.5%, *Daptrius chimachima*, Belo Horizonte, Minas Gerais, Brazil, March 2024) were deposited under the accession number UFMG-HEM-0060 (lineage 008).

***DNA sequences***: The partial mitochondrial cytochrome b fragment (480 bp) obtained corresponded in 100% identity to the lineage *Haemoproteus* sp. haplotype 46 (GenBank Acc. No. AF465594/POLPLA01 lineage from Crested Caracara, *C. plancus*). Mitochondrial partial *cytb* gene (POLPLA01 lineage) from *C. plancus* sample ID 050: 479 bp, Genbank Acc. No. PX209361 and *D. chimachima* sample ID 008: 479 bp, Genbank Acc. No. PX209362. Near-complete mitochondrial genome of POLPLA01 lineage from Crested Caracara, *Caracara plancus* ID 050: 5,455 bp, GenBank Acc. No. PX277568.

***Site of infection***: Mature erythrocytes.

***Etymology***: The specific epithet “trarotraro” comes from the indigenous Guarani name for the distinctive rattling call of the caracara. This sharp and evocative sound is often produced when the bird feels agitated, echoing across the open landscapes of South America. By using this ancestral term, the name honors both the cultural heritage and the unique presence of this species in its environment.

### Peripheral blood parasite morphometry

Erythrocytes infected by *H. trarotraro* n. sp. exhibited significant morphometric differences compared to uninfected cells. Erythrocyte length varied significantly among groups (Welch ANOVA: *F* = 16.545, *p* = 3.325 × 10^−^^6^), with both microgametocyte-infected (*p* < 0.00001) and macrogametocyte-infected cells (*p* = 0.0001) being longer than uninfected erythrocytes. No significant difference was found between the two infected groups (*p* = 0.9099). Erythrocyte area was significantly larger in parasitized cells (One-way ANOVA: *F* = 13.71, *p* = 7.01 × 10^−^^6^), with both microgametocyte (*p* = 0.000012) and microgametocyte (*p* = 0.0005) infected erythrocytes differing from uninfected cells, but not from each other (*p* = 0.7138). For the nuclear area, significant differences were found (One-way ANOVA: *F* = 8.014, *p* = 0.000653). Macrogametocyte-infected erythrocytes had smaller nuclear areas than both microgametocyte-infected (*p* = 0.0102) and uninfected cells (*p* = 0.00065), with no difference between microgametocyte-infected and uninfected erythrocytes (*p* = 0.6202).

Nuclear length (One-way ANOVA: *F* = 3.679, *p* = 0.0294) and width (Kruskal–Wallis: *χ*^2^ = 21, *p* < 0.0001) were also significantly different. Microgametocyte-infected erythrocytes exhibited significantly greater nucleus length compared to macrogametocyte-infected cells (*p* = 0.023), while no significant differences were observed between either infected group and uninfected erythrocytes (*p* > 0.1). Infected erythrocytes (both microgametocytes and macrogametocytes) showed greater nuclear widths than uninfected ones (*p* = 0.0043 and *p* < 0.00001, respectively), with no difference between the infected groups (*p* = 0.1413).

In the direct comparison between gametocytes, microgametocytes had significantly larger lengths (Mann–Whitney: *W* = 247.5, *p* = 0.013) and areas (Student’s t = −3.12, *p* = 0.0029) than macrogametocytes. Macrogametocytes exhibited higher NDR values than microgametocytes (Mann–Whitney: *W* = 561, *p* = 0.009613). No significant differences were found in width, number and length of hemozoin granules (all *p* > 0.05).

### Phylogenetic analyses

**Figure 2:**
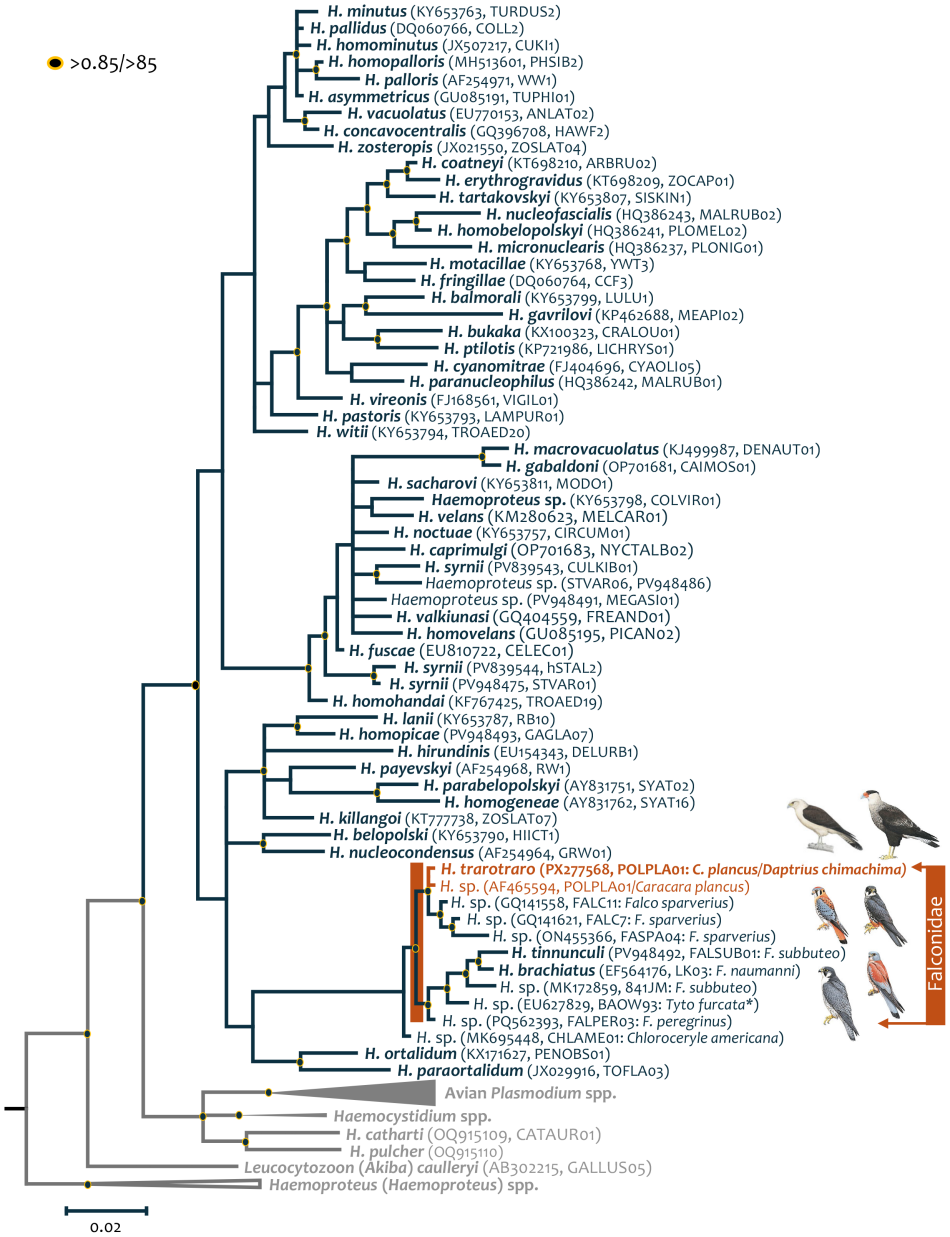
Bayesian phylogenetic hypothesis of *Haemoproteus trarotraro* n. sp. infecting *Caracara plancus* and *Daptrius chimachima* sampled in Brazil, based on partial parasite *cytb* gene (450 bp, excluding gaps). Nodes with posterior probabilities and bootstraps >0.85/ > 85 respectively are shown with a dot. Outgroups are indicated in grey. GenBank accession numbers and their lineage identifiers, as deposited in the MalAvi database, are provided in parenthesis for the sequences used in the analysis. *Haemoproteus trarotraro* n. sp. is written in orange.

**Figure 3:**
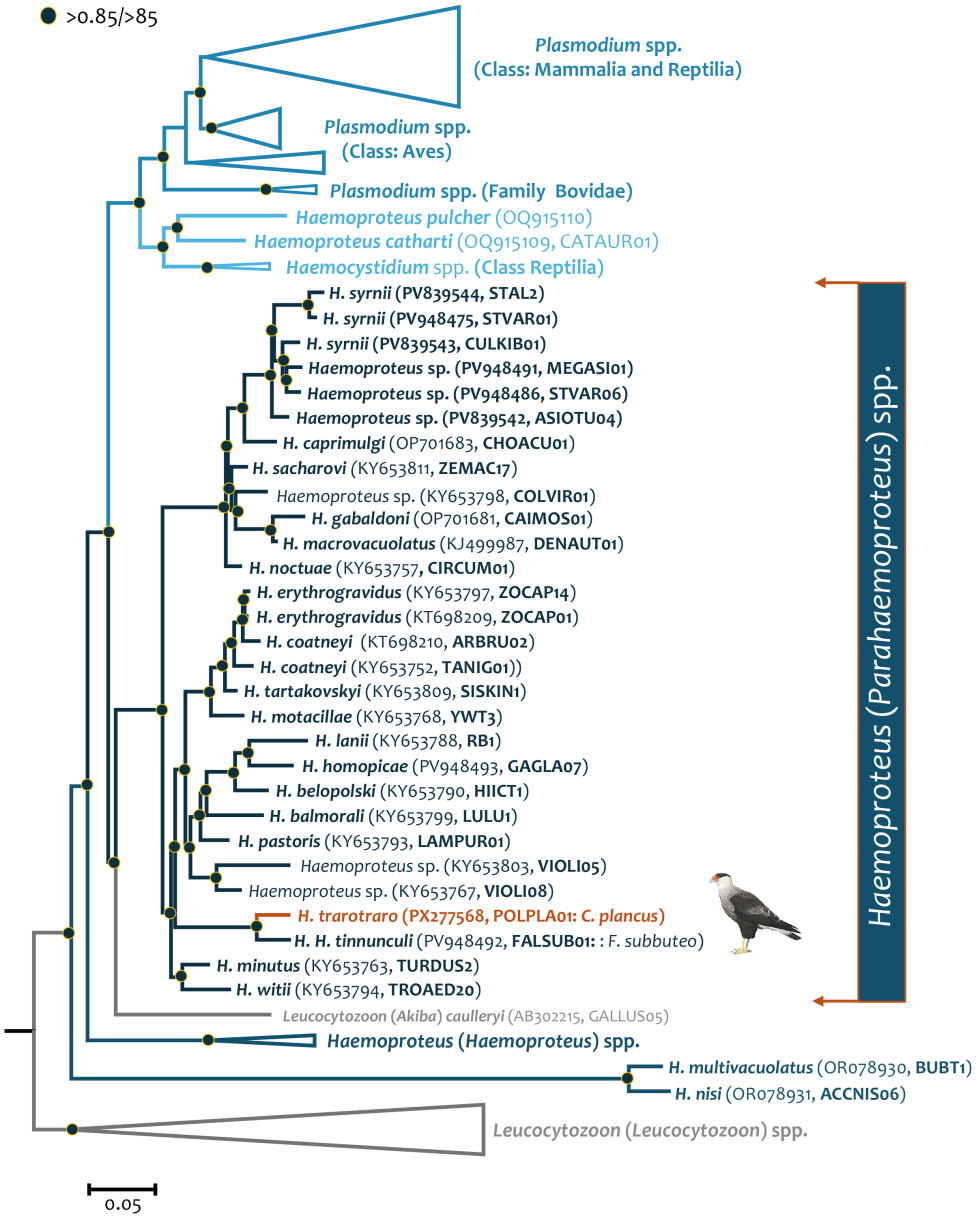
Bayesian phylogenetic hypothesis of *Haemoproteus trarotraro* n. sp. (lineage POLPLA01) infecting a Crested Caracara (*Caracara plancus*) sampled in Minas Gerais, Brazil, based on parasite near-complete mtDNA genome (5,082 bp, excluding gaps). Nodes with posterior probabilities and bootstraps >0.85/ > 85 respectively are shown with a dot. Outgroups are indicated in grey. GenBank accession numbers and their lineage identifiers, as deposited in the MalAvi database, are provided in parenthesis for the sequences used in the analysis. *Haemoproteus trarotraro* n. sp. is written in orange.

The *cytb* gene fragment-based phylogenetic reconstruction (Fig. 2) placed *H. trarotraro* n. sp. within an independent and strongly supported clade comprising *Haemoproteus* lineages from American Kestrels (*Falco sparverius*) sampled in the USA, including FALC7 (GenBank Acc. No. GQ141621), FALC11 (GenBank Acc. No. GQ141558), and FASPA04 (GenBank Acc. No. ON455366). In contrast, *H. tinnunculi* and *H. brachiatus* were recovered in a separate clade together with *Haemoproteus* lineages identified from the Eurasian Hobby (*Falco subbuteo*; GenBank Acc. No. MK172859) in France, the Barn Owl (*Tyto furcata*; GenBank Acc. No. EU627829), and the Peregrine Falcon (*Falco peregrinus*; GenBank Acc. No. PQ562393; Bell et al., 2025) in the USA. Although with a low node support (PP: 0.53, bootstrap: 61), both clades appeared sharing a common ancestor with *H. ortalidum* (KX171627, PENOBS01) from Dusky-legged Guan (*Penelope obscura*) and *H. paraortalidum* (JX029916, TOFLA03) from Ochre-lored Flatbill (*Tolmomyias flaviventris*), both bird samples collected in Brazil. Although there is not a mtDNA genome available for *H. brachiatus*, the close phylogenetic relationships between *H. trarotraro* n. sp. and *H. tinnunculi* based on their mtDNA is shown in Fig. 3.

Overall, the genetic distance between *H. tinnunculi* and *H. trarotraro* n. sp. based on partial *cytb* gene lineages and near-complete mtDNA genomes was 0.023 ± 0.007 (Table 3A) and 0.031 ± 0.002 (Table 3B), respectively. The genetic distance between *H. trarotraro* n. sp. and *H. brachiatus* was 0.018 ± 0.006 (Table 3A).

## Discussion

The classification and description of species within the Haemosporida order are complex and lack a straightforward criterion. The current approach involves using multiple lines of evidence, known as an integrative approach. This study contributes to the identification of a new *Haemoproteus* species found in Falconiformes: *Haemoproteus trarotraro* n. sp. This species appears to belong to a clade that includes several lineages primarily identified in other Falconiformes (Ricklefs & Fallon, 2002; Outlaw & Ricklefs, 2009; Outlaw & Ricklefs, 2010; Tinajero et al., 2019; Bell et al., 2025). Interestingly, some of these lineages have also been observed in birds from various orders (Ishak et al., 2008; Durrant et al., 2006; Fecchio et al., 2019). Although most cases lack evidence of host competence for sustaining transmission, the widespread occurrence of these lineages suggests that the host specificity of these parasites may be less strict at the order level than previously thought (Valkiūnas, 2005; Outlaw & Ricklefs, 2009; Gupta et al., 2019). For example, several case studies (Inumaru et al., 2020) and extensive investigations indicate that this could represent a broader trend among South American parasites (Moens & Pérez-Tris, 2016). In the latter study, molecular detection methods revealed that *Haemoproteus witti* is widely distributed across various bird species, implying that it may be a generalist. However, gametocytes—essential for transmission—are only present in hummingbirds, indicating a degree of host specialization at this stage. Therefore, it is crucial to determine how non-falconiform hosts may act as paratenic hosts, preventing the completion of the parasite’s life cycle. Although the presence of gametocytes has not been conclusively linked to specific molecular lineages in many reported hosts, their common occurrence suggests that at least some of these hosts may be capable of supporting the parasite’s full cycle. This underscores the importance of integrating both molecular and microscopic approaches to accurately assess host-parasite relationships on local and global scales.

The two species thus far recognized as infecting Falconiformes, *H. brachiatus* and *H. tinnunculi*, exhibit a genetic distance of only 0.009 at the *cytb* gene level (see Table 3), differing by just four synonymous substitutions. Moreover, both species were described from hosts of the same genus and occur in overlapping geographic regions. Nevertheless, these parasites are treated as distinct species, primarily based on subtle morphological characters that may fall within the spectrum of intraspecific variation commonly observed in haemosporidian parasites (Garnham, 1966; Laird & Van Riper, 1981; Valkiūnas, 2005; Vanstreels et al., 2014a; Vieira et al., 2025a). Both species are characterized by the ability of their fully developed gametocytes to encircle the parasite’s nucleus completely. However, developing gametocytes of *H. brachiatus* are typically highly irregular or amoeboid in shape, whereas those of *H. tinnunculi* tend to be more uniform. In addition, advanced gametocytes of *H. brachiatus* frequently do not maintain close adherence to the erythrocyte envelope, in contrast to *H. tinnunculi*, whose gametocytes usually remain tightly associated with the host cell membrane throughout development (Valkiūnas, 2005; Valkiūnas et al., 2019).

**Table 3:** Genetic distance and standard error of *Haemoproteus* lineages based on (A) partial *cytb* gene (450 bp) and (B) mtDNA genome (5,424 bp excluding gaps). The number of base substitutions per site between pair of sequences are shown. Standard error estimate(s) are shown above the diagonal. Numbers in bold correspond to the pairwise distance estimated between *H. trarotraro* n. sp. and closely related parasites. Analyses were conducted using the Tajima-Nei model [1]. The rate variation among sites was modeled with a gamma distribution (shape parameter = 1). Evolutionary analyses were conducted in MEGA7 [3]. Parasites sequences correspond to Falconiformes except those with an “*” (*Tyto furcata*: Strigiformes/Tytonidae, *Chloroceryle americana*: Coraciiformes/Alcedinidae).

	**(A)**	**Genetic distance (Standard Error)**										
	***Haemoproteus*** ***cytb*** ** gene lineage**	**1**	**2**	**3**	**4**	**5**	**6**	**7**	**8**	**9**	**10**	**11**
**1**	***H. trarotraro*** (PX209361/PX209362, POLPLA01: *Caracara plancus/Daptrius chimachima* )		0.000	0.002	0.003	0.005	0.007	0.006	0.007	0.005	0.003	0.003
**2**	*Haemoproteus* sp. (AF465594, POLPLA01/*C. plancus*)	**0.000**		0.002	0.003	0.005	0.007	0.006	0.007	0.005	0.003	0.003
**3**	*Haemoproteus* sp. (GQ141558, FALC11: *Falco sparverius*)	**0.002**	0.002		0.002	0.005	0.007	0.007	0.007	0.005	0.004	0.004
**4**	*Haemoproteus* sp. (GQ141621, FALC7: *F. sparverius*)	**0.004**	0.004	0.002		0.004	0.007	0.006	0.007	0.005	0.004	0.004
**5**	*Haemoproteus* sp. (ON455366, FASPA04: *F. sparverius*)	**0.014**	0.014	0.011	0.009		0.008	0.007	0.008	0.006	0.005	0.005
**6**	***H. tinnunculi*** (PV948492, FALSUB01: *F. subbuteo*)	**0.023**	0.023	0.025	0.023	0.028		0.004	0.006	0.006	0.006	0.007
**7**	***H. brachiatus*** (EF564176, LK03: *F. naumanni*)	**0.018**	0.018	0.021	0.018	0.023	0.009		0.005	0.005	0.006	0.006
**8**	*Haemoproteus* sp. (MK172859, 841JM: *F. subbuteo*)	**0.021**	0.021	0.023	0.021	0.025	0.016	0.011		0.006	0.006	0.007
**9**	*Haemoproteus* sp. (EU627829, BAOW93: Tyto furcata*)	**0.014**	0.014	0.014	0.011	0.016	0.018	0.014	0.016		0.004	0.005
**10**	*Haemoproteus* sp. (PQ562393, FALPER03: *F. peregrinus*)	**0.004**	0.004	0.007	0.009	0.014	0.018	0.014	0.016	0.009		0.003
**11**	*Haemoproteus* sp. (MK695448, CHLAME01: Chloroceryle americana*)	**0.004**	0.004	0.007	0.009	0.014	0.023	0.018	0.021	0.014	0.004	
	**(B)**	**GD (SE)**										
	***Haemoproteus*** ** mtDNA genome lineage**	**1**	**2**									
**1**	***H. trarotraro*** n. sp. (PX277568, POLPLA01: *C. plancus/D. chimachima*)		0.002									
**2**	***H. tinnunculi*** (PV948492, FALSUB01: *F. subbuteo*)	**0.031**										

Although a divergence of approximately 5% in the partial *cytb* gene, is generally considered reliable to support species when compared to others that share a recent common ancestor, a genetic distance is not a taxonomic trait. Furthermore, when morphological data indicate differences, new species have been proposed (Levin et al., 2012; Valkiūnas et al., 2019), suggesting that morphological evidence is favored to propose a new species over the alternative hypothesis that limited molecular divergences in the presence of distinct morphological characteristics may indicate phenotypic plasticity (Pacheco & Escalante, 2020). It is also important to realize that *cytb* gene exhibits a slower rate of evolution in *Haemoproteus* when compared to other Haemosporida (Pacheco et al., 2018a), making the use of genetic “distances” even more complex. Nevertheless, following the standard practice in the field, *H. trarotraro* n. sp. shows a genetic divergence of 2.3 to 3.1% when compared to *H. tinnunculi* based on the partial *cytb* gene sequences and mtDNA genomes, respectively, and 1.8% from *H. brachiatus* based on the partial *cytb* gene (see Table 3). The *cytb* gene fragment shows 9–12 nucleotide substitutions among the three species, including one non-synonymous change (see Table 4).

**Table 4:** Position and classification of mutations between *H. brachiatus*/*H. tinnunculi* and *H. trarotraro* n. sp. at the *cytb* gene level. A.A. = aminoacids.

**Codon position**	**Codon** ***H. brachiatus*** **/** ***H. tinnunculi***	**A.A.** ***H. brachiatus*** **/** ***H. tinnunculi***	**Códon** ***H. trarotraro*** ** n. sp.**	**A.A.** ***H. trarotraro*** ** n. sp.**	**Mutation**
79	GCC	A	GCT	A	Synonym
81	GGT	G	GGC	G	Synonym
85	GTC	V	GTA	V	Synonym
86	TTT	F	TTC	F	Synonym
106	CTA	L	TTA	L	Synonym
113	GTT	V	ATT	I	Non-synonym
116	CTA	L	TTA	L	Synonym
139	ACA	T	ACT	T	Synonym
149	CCA	P	CCT	P	Synonym
181	CTA	L	TTA	L	Synonym
198	AAC	N	AAT	N	Synonym
236	GGA	G	GGT	G	Synonym

It is worth noting that *H. brachiatus*, *H. tinnunculi*, and their associated lineages have been reported mainly from species of the genus *Falco* in Eurasia (Valkiūnas, 2005; Valkiūnas et al., 2019), whereas *H. trarotraro* n. sp. has been recorded only in the Americas, predominantly associated with *C. plancus* (Ricklefs & Fallon, 2002) and *F. sparverius* (Outlaw & Ricklefs, 2009; Outlaw & Ricklefs, 2010; Tinajero et al., 2019). Although Foster et al. (1998) reported a high prevalence of *H. tinnunculi* in Crested Caracaras in Florida, their data were based solely on morphological evaluation, which limits the reliability of the identification.

The morphological analysis of gametocytes from *H. trarotraro* n. sp. indicates that none of the specimens observed across all sampled slides and time points fully encircle the host cell nucleus. This notable trait distinctly sets *H. trarotraro* n. sp. apart from related species *H. tinnunculi* and *H. brachiatus*, both of which exhibit complete encirclement. Furthermore, macrogametocytes of *H. trarotraro* n. sp. exhibit multiple small vacuoles, a trait not reported in either of the other two species. These observations are consistent with the phylogenetic hypothesis presented in this study using *cytb* gene sequences, in which *H. trarotraro* n. sp. was not recovered as the sister taxon of *H. tinnunculi* or *H. brachiatus* (Fig. 2). Instead, it was placed within an independent clade that includes lineages sampled from American Kestrels (*Falco sparverius*) from USA (Outlaw & Ricklefs, 2009; Keith et al., 2022).

Like in other Haemosporida, additional sampling of Falconiformes in both Eurasia and the Americas is needed to understand genetic divergence patterns, as it is challenging to associate genetic structures or phenotypic discontinuities with species in the absence of an understanding of the process that gave rise to them (speciation). For example, limited molecular divergence among species in parasite clades may be the result of rapid speciation leading to clear phenotypic divergence (*e.g.*, Muehlenbein et al., 2014). In contrast, in other cases, genetic divergences could be the result of geographic or host structure that limits gene flow. Ultimately, what is considered a species depends on how the proposed taxa integrates multiple lines of information that consistently make predictions about its biology (*e.g.*, host range, distribution, overall ecology, and other phenotypes) with the prevailing trend of using morphological characteristics for delimiting species in the traditional Haemosporida taxonomy (Outlaw & Ricklefs, 2014). As such, *H. trarotraro* n. sp., meets the standard as it links morphological and molecular evidence that make it distinct from other known accepted species.

The most robust practice appears to be evaluating geographic and host-related factors, the nature of mutations on those loci used as diagnostic markers (*e.g.*, synonymous *vs.* nonsynonymous when using molecular markers such as the partial *cytb* gene), and morphological divergence whenever possible (Hellgren et al., 2007; Valkiūnas et al., 2009; Outlaw & Ricklefs, 2014). Furthermore, exploring near-complete mitochondrial genomes enriches the analysis by providing a greater number of informative sites, thereby allowing the reconstruction of more robust phylogenies (Pacheco et al., 2018b). Although the mtDNA database for haemosporidians is still in its early stages of development, it is steadily expanding. In this study, we were able to compare the mitochondrial genomes of *H. trarotraro* n. sp. and *H. tinnunculi*. Given the available data, *H. trarotraro* n. sp. and *H. tinnunculi* appeared to share a common ancestor (see Fig. 3).

Given the division of *C. plancus* into two subspecies with distinct geographic distributions, it is plausible to hypothesize that the hemoparasite fauna of *C. plancus cheriway* differs from that of *C. plancus plancus*, due to geographic barriers and differences in vector distribution (Fecchio et al., 2017; Fecchio et al., 2018; Doussang et al., 2019). However, the *H. trarotraro* n. sp. lineage recovered in this study from a *C. plancus plancus* individual in Brazil is identical to one previously found in *C. plancus cheriway* in Florida, USA. While a historical scenario of allopatric divergence has been proposed for these host subspecies, current evidence indicates a broad zone of overlap and potential intergradation across the Amazon basin (Dove & Banks, 1999; Morrison & Dwyer, 2021). This situation could facilitate parasite exchange between the two subspecies within that specific region, resembling the ‘corridor hypothesis’ proposed by Pacheco et al. (2019), which supports the genetic flow of *P. vivax* populations between northwestern Colombia and the South Pacific coast.

Nonetheless, we should not dismiss alternative transmission scenarios. One possibility worth considering is the involvement of other bird species as bridge reservoirs (Moens & Pérez-Tris, 2016; Doussang et al., 2021), such as the Yellow-headed Caracara. As reported here, an individual of this species was also found to harbor the same lineage identified in Crested Caracaras. Although not naturally occurring in the United States, occasional records of *D. chimachima* have been reported, including in Florida (American Birding Association, 2023; Fuchs, Johnson & Mindell, 2015) highlighted this species’ rapid diversification and its ability to exploit anthropogenically altered environments, particularly deforested areas converted into cattle pastures. This should also serve as a warning for potential pathogen spillover, warranting further investigation, especially due to the likely generalist behavior of *H. trarotraro* n. sp., which may promote an amplifying effect on infection (Moens & Pérez-Tris, 2016; Doussang et al., 2021). Considering the high parasitemias of mature macro- and microgametocytes observed in this study, the hypothesis of parasite spillover to competent vectors—and subsequently to other hosts—is entirely plausible.

## Conclusions

*Haemoproteus trarotraro* n. sp. represents the third valid species described in the order Falconiformes, and the first reported from South America (Brazil). Although it shows only ∼2% *cytb* gene divergence from *H. tinnunculi* and *H. brachiatus*, its notable morphological differences, occurrence in distinct host species with different geographic ranges, and the results of both partial *cytb* gene and mtDNA phylogenetic analyses collectively support the recognition of *H. trarotraro* n. sp. as a new species. From this, we may also discuss the hypothesis that small genetic distances in the partial *cytb* gene are a common feature of distinct *Haemoproteus* species parasitizing Falconiformes. While *Haemoproteus* has traditionally been considered host-specific at the avian order level, lineages likely associated with *H. trarotraro* n. sp. have already been detected in multiple bird orders across the Americas. Accurately identifying these lineages, along with their morphological traits, host associations, and geographic distribution essential for enhancing our understanding of their evolutionary history and zoogeographical patterns. Moreover, the description of *H. trarotraro* n. sp. in two host species commonly admitted to a wildlife rehabilitation center warrants attention, particularly due to the potential for spillover into endangered, susceptible and/or endemic bird species. Assessing the pathogenic potential of this new parasite, alongside systematic haemosporidian screening in captive bird populations, is essential, especially when individuals are intended for reintroduction or housed near threatened species.

##  Supplemental Information

10.7717/peerj.20653/supp-1 Supplemental Information 1 mtDNA sequence of *Haemoproteus (Parahaemoproteus) trarotraro* n. sp. (Genbank Acc. No. PX277568)

10.7717/peerj.20653/supp-2 Supplemental Information 2 Gametocytes of *Haemoproteus trarotraro* n. sp. from the blood of Yellow-headed Caracara (*Daptrius chimachima*) sampled in Minas Gerais, Brazil (A-K) Young gametocytes. (L-R) Macrogametocytes. (S-T) Microgametocytes. Black arrowheads: haemozoin granules; black long arrows: vacuoles; white arrowheads: volutin. Giemsa-stained thin blood films. Scale bar = 10 µm.

## Additional Information and Declarations

### Field Study Permissions

The following information was supplied relating to field study approvals (*i.e.*, approving body and any reference numbers): The collections within CETAS-BH were approved by the Instituto Estadual de Florestas—IEF under the Authorization No. 75722467 (Process No. 2100.01.0035718/2023-91) and by the Ethics Committee on Animal Experimentation (CETEA) of the Universidade Federal de Minas Gerais, Brazil (Protocol No. 48/2024).

### Data Availability

The following information was supplied regarding data availability: The raw data are available in the Supplemental File. The sequences are available at GenBank: PX209362, PX209361 and PX277568.

### New Species Registration

The following information was supplied regarding the registration of a newly described species: Publication LSID: urn:lsid:zoobank.org:pub:E3DAA6A6-1060-4192-A64E-B734CAB9C97C. *Haemoproteus trarotraro* Species LSID: urn:lsid:zoobank.org:act:99F4B911-448E-4FBF-861A-A6D2690A3C5C.
