# Supplementary figures and images for: Morphological description and near-complete mitochondrial genome of Haemoproteus (Parahaemoproteus) trarotraro n. sp.: a widely distributed species reported in Brazilian falcons
Source: PeerJ. 2026 Feb 24;14:e20653. doi: 10.7717/peerj.20653 (PMC12947763; doi:10.7717/peerj.20653)

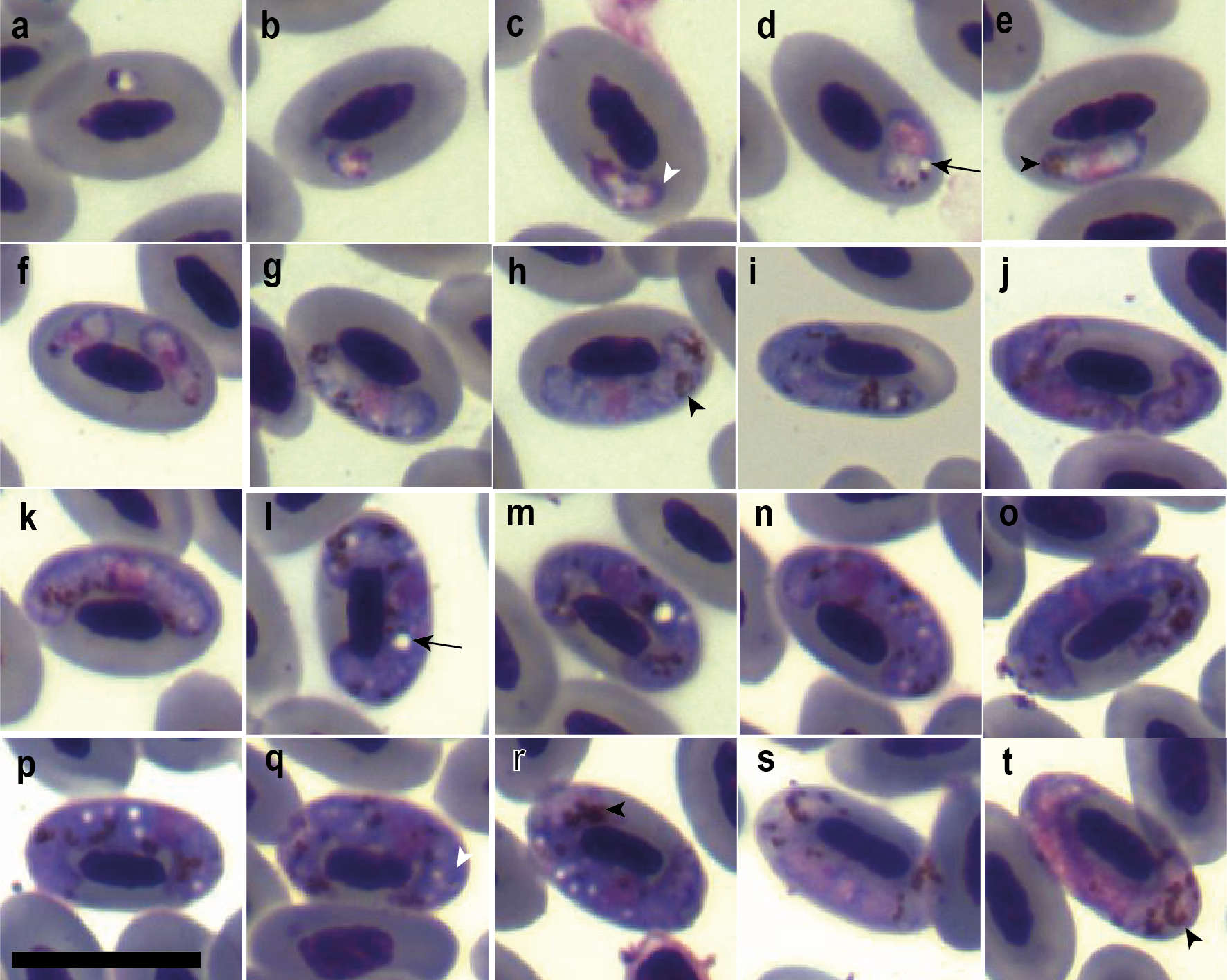

Supplement: Supplemental Information 2 — (A-K) Young gametocytes. (L-R) Macrogametocytes. (S-T) Microgametocytes. Black arrowheads: haemozoin granules; black long arrows: vacuoles; white arrowheads: volutin. Giemsa-stained thin blood films. Scale bar = 10 µm. [file peerj-14-20653-s002.png]
